# Screening rates for HIV and diabetes in patients with active TB: results of a nationwide survey in Japan

**DOI:** 10.5588/ijtldopen.24.0282

**Published:** 2024-07-01

**Authors:** Y. Kimura, Y. Sasabuchi, T. Jo, Y. Hashimoto, R. Kumazawa, M. Ishimaru, H. Matsui, A. Yokoyama, G. Tanaka, H. Yasunaga

**Affiliations:** ^1^ Department of Clinical Epidemiology and Health Economics, School of Public Health, The University of Tokyo, Tokyo, Japan;; ^2^ Clinical Research Center, NHO Tokyo National Hospital, Tokyo, Japan;; ^3^ Department of Real-world Evidence, Graduate School of Medicine, The University of Tokyo, Tokyo, Japan;; ^4^ Department of Respiratory Medicine, The University of Tokyo, Tokyo 113-0033, Japan;; ^5^ Save Sight Institute, The University of Sydney, Sydney, NSW, Australia;; ^6^ Department of Public Health and Epidemiology, Meiji Pharmaceutical University, Tokyo, Japan;; ^7^ Institute of Education, Tokyo Medical and Dental University (TMDU), Tokyo, Japan;; ^8^ Division for Health Service Promotion, The University of Tokyo, Tokyo, Japan

**Keywords:** human immunodeficiency virus, diabetes mellitus, epidemiology, national administrative database, TB

Dear Editor,

The probability of developing TB disease is much higher among people living with HIV and for people with risk factors such as diabetes mellitus (DM).^1,2^ Comorbidity with HIV or DM also leads to higher rates of morbidity and mortality in patients with TB.^1^ Therefore, WHO issued policy guidelines on collaborative TB-HIV activities and a collaborative framework for TB and DM, including screening for HIV and DM in patients with TB disease.^3,4^ Understanding the actual prevalence of HIV and DM and the performance rates of screening in patients with TB disease is essential for public health. In Japan, the Infectious Diseases Control Law mandates comprehensive reporting and monitoring of cases of TB disease, and all are registered in the Japan Tuberculosis Surveillance (JTBS) system.^5^ Although the JTBS system has served as a vital resource for understanding the landscape of TB and informing health policy decisions, it has limitations regarding comorbidities and tests. Comorbidity information is restricted to self-reported data on DM and tests are limited to HIV testing, chest radiography and bacteriological examinations. Furthermore, the JTBS annual report indicates a high percentage (over 90%) of cases for which HIV testing is unknown.^5^ Thus, data on the actual prevalence of comorbidity for HIV, DM and TB are not available. We therefore, aimed to assess these data using a national administrative database covering 99% of the hospitals in Japan.^6^

This was a cross-sectional study from 2016 to 2021 using Japan's National Database of Health Insurance Claims and Specific Health Checkups (NDB).^6,7^ We defined patients with TB in each year as those who met the following two criteria: 1) those with diagnostic codes for TB (see Supplementary Table S1) in that year and without any TB diagnostic codes in the 2 years up to their initial diagnosis, and 2) those who were started on treatment for TB including isoniazid. The rationale for these case definitions is provided in the Supplementary Data. We clarified comorbidity for HIV and DM and the screenings for these conditions, which were determined according to the information from the month of TB diagnosis and the 11 months prior to this. The comorbidities and tests were identified using claims with the corresponding diagnostic (Supplementary Data Table S1) and test codes. The screening rates based on other timeframes and facilities where physicians diagnosed TB were also clarified. Further explanations are provided in the Supplementary Data. As there are no validation studies for the NDB as of January 2024,^8^ we confirmed the validity of our case definitions by comparing the number of patients with TB reported by the JTBS system (assumed to be the true number of patients with TB in Japan) and the number of patients based on our case definitions for each year. All analyses were performed using PostgreSQL v15 (https://www.postgresql.org/) and Python v3.7 (https://www.python.org/). This study was approved by the Institutional Review Board of the University of Tokyo, Tokyo, Japan. The requirement for informed consent was waived due to the anonymous nature of the database.

The prevalence of TB-HIV comorbidity remained consistent from 2016 to 2021, with values of 0.4%, 0.4%, 0.4%, 0.3%, 0.3%, and 0.3% for each year. In contrast, the prevalence of TB-DM comorbidity increased during the same period, with values of 18.8%, 20.0%, 21.5%, 21.7%, 23.1%, and 24.7% for each year. The Table shows the results of the tests that patients with TB underwent. The rate of HIV screening in the month of TB diagnosis (and the 11 months prior) remained stable, ranging from 12.1% to 13.5%. The frequency of DM screening tests over the same period increased slightly from 91.3% to 93.6%. When the timeframe was altered from 12 months to either 3 months or 1 month, or when the analyses were limited to facilities that diagnosed TB, the observed trends remained consistent. In 2016–2021, respectively 19,775, 18,537, 17,181, 15,975, 13,370, and 12,196 cases of TB were identified according to our case definition, whereas fewer cases were reported in the JTBS system (respectively 17,625, 16,789, 15,590, 14,460, 12,739, and 11,519). The percentage differences were 12.2%, 10.4%, 10.2%, 10.5%, 5.0%, and 5.9%, respectively.

**Table.** Tests for HIV and DM in patients with TB in Japan.

	2016	2017	2018	2019	2020	2021
	*n* (%)	*n* (%)	*n* (%)	*n* (%)	*n* (%)	*n* (%)
All cases	19,775 (100.0)	18,537 (100.0)	17,181 (100.0)	15,975 (100.0)	13,370 (100.0)	12,196 (100.0)
HIV screening within 1 month	1,450 (7.3)	1,510 (8.2)	1,357 (7.9)	1,308 (8.2)	1,041 (7.8)	951 (7.8)
DM screening within 1 month	14,843 (75.1)	14,087 (76.0)	13,192 (76.8)	12,264 (76.8)	10,487 (78.4)	9,567 (78.4)
HIV screening within 3 months	1,953 (9.9)	2,008 (10.8)	1,836 (10.7)	1,750 (11.0)	1,404 (10.5)	1,292 (10.6)
DM screening within 3 months	16,993 (85.9)	16,060 (86.6)	15,034 (87.5)	13,912 (87.1)	11,855 (88.7)	10,850 (89.0)
HIV screening within 12 months	2,398 (12.1)	2,429 (13.1)	2,239 (13.0)	2,156 (13.5)	1,719 (12.9)	1,577 (12.9)
DM screening within 12 months	18,063 (91.3)	16,983 (91.6)	15,865 (92.3)	14,683 (91.9)	12,506 (93.5)	11,413 (93.6)
Tests performed in the facility which diagnosed TB						
HIV screening within 1 month	1,054 (5.3)	1,090 (5.9)	982 (5.7)	955 (6.0)	771 (5.8)	687 (5.6)
DM screening within 1 month	13,063 (66.1)	12,383 (66.8)	11,631 (67.7)	10,790 (67.5)	9,233 (69.1)	8,414 (69.0)
HIV screening within 3 months	1,443 (7.3)	1,463 (7.9)	1,355 (7.9)	1,302 (8.2)	1,069 (8.0)	940 (7.7)
DM screening within 3 months	15,162 (76.7)	14,260 (76.9)	13,389 (77.9)	12,426 (77.8)	10,566 (79.0)	9,696 (79.5)
HIV screening within 12 months	1,733 (8.8)	1,704 (9.2)	1,596 (9.3)	1,537 (9.6)	1,264 (9.5)	1,112 (9.1)
DM screening within 12 months	16,150 (81.7)	15,166 (81.8)	14,175 (82.5)	13,201 (82.6)	11,245 (84.1)	10,288 (84.4)

DM = diabetes mellitus.

A positivity rate of 0.2–0.3% for HIV in patients with TB was reported in the JTBS system, but over 90% of the test results were unknown, and only 7.4% of the results in 2021 were explicitly positive or negative.^5^ The low HIV screening rate of 12–14% in our study indicates that the ‘unknown’ category in the JTBS system likely represents tests not performed, suggesting potential under-detection of HIV in patients with TB. Globally, the WHO and the Centers for Disease Control and Prevention recommend HIV screening among patients with TB.^3,9^ HIV screening for all patients with TB is recommended in 85% of European countries, including those with low HIV incidence.^10^ However, in Japan, there are no clear national guidelines specifically recommending HIV screening for patients with TB. Because early initiation of combined antiretroviral therapy is a priority for HIV-infected patients with TB,^1^ it is essential to consider these international recommendations and construct a programme that promotes HIV screening in Japan.

Performance rates for DM screening were >90% during the study period, indicating low oversight in patients with TB comorbidity. There was a discrepancy between the DM prevalence in our study (18.8–24.7%) and that reported in the JTBS system (14.2–15.4%). The discrepancy may be because our data are dependent on claims data vs. the JTBS system, which uses self-reported data. Although the specific reasons for the increase in DM could not be clarified in this study, one possible explanation is that the number of patients with TB risk factors, such as DM, did not decrease as much as the number of patients without these risk factors. This discrepancy may have led to a rising proportion of patients with these risk factors over time.

A limitation of our study is that the diagnostic codes for TB and their combination with treatments (including isoniazid) have not been validated due to the restrictions on linking the NDB to other databases.^8^ However, based on our case definition, the number of patients is closely aligned with that of the JTBS, indicating that diagnostic codes and medications from the NDB can be used to identify patients accurately.

Using a national administrative database, we have shown that the performance rate of HIV screening tests was low (ranging between 12.1% and 13.5%), whereas DM screening rates were high (over 90%). It is therefore essential to enhance our capacity for HIV screening of patients with TB in Japan. Additionally, combining the JTBS system with claims data would allow for rapid monitoring of screening test results.

## Supplementary Material


