# Supplementary material for: Screening rates for HIV and diabetes in patients with active TB: results of a nationwide survey in Japan
Source: IJTLD Open. 2024 Jul 1;1(7):326–8. doi: 10.5588/ijtldopen.24.0282 (PMC11257088; doi:10.5588/ijtldopen.24.0282)
Supplement: Supplementary file 1 [file ijtldopen0282_supplementarydata1.docx]

©2024 The Authors http://dx.doi.org/10.5588/ijtldopen.24.0282 **SUPPLEMENTARY DATA**

**Screening rates for HIV and diabetes in patients with active TB: results of a nationwide survey in Japan**

**The case definition**

We defined patients with tuberculosis disease in each year (from 2016 to 2021) as those who met the following two criteria: 1) those with diagnostic codes for tuberculosis disease (Table S1) in that year and without any tuberculosis disease diagnostic codes in the 2 years up to their initial diagnosis in that year, and 2) those who were started on chemotherapy including isoniazid. We used this case definition for the following reasons: First, because the diagnostic codes for tuberculosis disease may persist even after a patient is cured, they may not indicate ~~active~~ tuberculosis disease. Second, patients who have completed tuberculosis disease treatment are typically monitored for the subsequent 2 years, and this period is associated with a higher risk of recurrence than the later years.^1^ Hence, we established a 2-year timeframe because patients with diagnostic codes for tuberculosis disease during the 2 years prior to their initial diagnosis in the year were likely to be under surveillance for recurrence. Third, approximately 95% of the patients with tuberculosis disease initiated chemotherapy including isoniazid, with the remaining patients receiving no treatment or almost none receiving chemotherapy without isoniazid.^2^ Furthermore, a subset of patients underwent isoniazid monotherapy, owing to the physicians’ concerns regarding adverse events associated with the initiation of multiple anti-tuberculosis medications.

**Identification of screening tests for HIV and diabetes**

We identified screening tests for HIV and diabetes based on the 12-month period prior to diagnosis. The 12-month periods included the diagnosis month of tuberculosis disease and the 11 months prior. Consequently, this identification method included screening tests conducted in the diagnosis month (including a certain period from the diagnosis date). It is reasonable to assume that physicians would perform screening tests for HIV or diabetes at the time of diagnosing patients with tuberculosis disease. However, if recent screening results are available, physicians may avoid repeating the screening tests. Therefore, a 12-month timeframe up to the month of diagnosis was adopted to perform the tests. However, to better assess the rate of screening closest to the tuberculosis disease diagnosis, we also examined the results after altering the timeframe from 12 months to 3 months or 1 month. Furthermore, we exclusively assessed the results from facilities where physicians diagnosed tuberculosis disease.

**References**

1 Qiu B, et al. Risk factors for types of recurrent tuberculosis (reactivation versus reinfection): A global systematic review and meta-analysis. Int J Infect Dis 2022; 116: 14-20.

2 TUBERCULOSIS IN JAPAN ANNUAL REPORT - 2022. https://jata.or.jp/english/dl/pdf/TB_in_Japan_2022FINrev.pdf; 2022 Accessed 15.01.2024.

**Supplementary Table S1. List of diagnostic codes**

| Diagnostic code | Corresponding ICD-10 code | Description | Our category |
| --- | --- | --- | --- |
| 8843756 | A150 | Pulmonary tuberculosis, confirmed by microscopy | Tuberculosis disease |
| 8843758 | A151 | Pulmonary tuberculosis, confirmed by culture only | Tuberculosis disease |
| 8843757 | A152 | Pulmonary tuberculosis, histologically confirmed | Tuberculosis disease |
| 8843685 | A154 | Intrathoracic lymph node tuberculosis, bacteriologically confirmed | Tuberculosis disease |
| 8843686 | A154 | Intrathoracic lymph node tuberculosis, histologically confirmed | Tuberculosis disease |
| 8849094 | A155 | Bronchial tuberculosis, bacteriologically confirmed | Tuberculosis disease |
| 8849095 | A155 | Bronchial tuberculosis, histologically confirmed | Tuberculosis disease |
| 8843704 | A156 | Tuberculous pleurisy, bacteriologically confirmed | Tuberculosis disease |
| 8843705 | A156 | Tuberculous pleurisy, histologically confirmed | Tuberculosis disease |
| 00116001 | A162 | Caseous pneumonia | Tuberculosis disease |
| 00119019 | A162 | Tuberculoma of lung | Tuberculosis disease |
| 8831388 | A162 | Active pulmonary tuberculosis | Tuberculosis disease |
| 8833037 | A162 | Tuberculous hemoptysis | Tuberculosis disease |
| 8833040 | A162 | Tuberculous bronchiectasis | Tuberculosis disease |
| 8833041 | A162 | Tuberculous pneumothorax | Tuberculosis disease |
| 8833042 | A162 | Tuberculous cavity | Tuberculosis disease |
| 8833073 | A162 | Tuberculous pulmonary fibrosis | Tuberculosis disease |
| 8833074 | A162 | Tuberculous lung abscess | Tuberculosis disease |
| 8833126 | A162 | Nodular pulmonary tuberculosis | Tuberculosis disease |
| 8833351 | A162 | Sclerotic pulmonary tuberculosis | Tuberculosis disease |
| 8838803 | A162 | Pneumonic tuberculosis | Tuberculosis disease |
| 8838810 | A162 | Pulmonary tuberculosis | Tuberculosis disease |
| 8838906 | A162 | Hilus tuberculosis | Tuberculosis disease |
| 0121005 | A163 | Hilar lymph node tuberculosis | Tuberculosis disease |
| 0123002 | A164 | Laryngeal tuberculosis | Tuberculosis disease |
| 8832151 | A164 | Tracheal tuberculosis | Tuberculosis disease |
| 8832161 | A164 | Bronchial tuberculosis | Tuberculosis disease |
| 0120002 | A165 | Tuberculous pleurisy | Tuberculosis disease |
| 8833043 | A165 | Tuberculous hemothorax | Tuberculosis disease |
| 8833067 | A165 | Tuberculous pyothorax (empyema) | Tuberculosis disease |
| 0109003 | A167 | Primary tuberculosis | Tuberculosis disease |
| 8833033 | A167 | Tuberculous primary infection | Tuberculosis disease |
| 4782047 | A168 | Pharyngeal peritonsillar abscess | Tuberculosis disease |
| 8830655 | A168 | Pharyngeal tuberculosis | Tuberculosis disease |
| 8835272 | A168 | Mediastinal tuberculosis | Tuberculosis disease |
| 8839441 | A168 | Nasopharyngeal tuberculosis | Tuberculosis disease |
| 8839717 | A168 | Sinus tuberculosis | Tuberculosis disease |
| 0119003 | A169 | Tuberculoma | Tuberculosis disease |
| 0188002 | A169 | Tuberculous fibrosis | Tuberculosis disease |
| 8833031 | A169 | Tuberculosis | Tuberculosis disease |
| 8833039 | A169 | Tuberculous cough | Tuberculosis disease |
| 8833047 | A169 | Tuberculous sclerosis | Tuberculosis disease |
| 8833072 | A169 | Tuberculous abscess | Tuberculosis disease |
| 8833075 | A169 | Tuberculous fever | Tuberculosis disease |
| 8838365 | A169 | Intractable tuberculosis | Tuberculosis disease |
| 8847112 | A169 | Multidrug-resistant tuberculosis | Tuberculosis disease |
| 0130001 | A170 | Tuberculous meningitis | Tuberculosis disease |
| 8832811 | A170 | Tuberculous arachnoiditis | Tuberculosis disease |
| 8833050 | A170 | Tuberculous pachymeningitis | Tuberculosis disease |
| 8833066 | A170 | Tuberculous leptomeningitis | Tuberculosis disease |
| 8836001 | A170 | Spinal meningitis tuberculosis | Tuberculosis disease |
| 8838735 | A170 | Tuberculous meningoencephalitis | Tuberculosis disease |
| 8835796 | A171 | Tuberculous meningioma | Tuberculosis disease |
| 8833062 | A178 | Tuberculous polyneuropathy | Tuberculosis disease |
| 8833069 | A178 | Tuberculous encephalomyelitis | Tuberculosis disease |
| 8833071 | A178 | Tuberculous brain abscess | Tuberculosis disease |
| 8835983 | A178 | Spinal tuberculosis | Tuberculosis disease |
| 8835984 | A178 | Spinal tuberculoma | Tuberculosis disease |
| 8838699 | A178 | Cerebral tuberculosis | Tuberculosis disease |
| 8838700 | A178 | Cerebral tuberculoma | Tuberculosis disease |
| 8834937 | A179 | Tuberculosis of the nervous system | Tuberculosis disease |
| 8831329 | A180 | Tuberculosis of the shoulder joint | Tuberculosis disease |
| 8831594 | A180 | Joint tuberculosis | Tuberculosis disease |
| 8833038 | A180 | Tuberculous synovitis | Tuberculosis disease |
| 8833044 | A180 | Tuberculous tenosynovitis | Tuberculosis disease |
| 8833051 | A180 | Tuberculous osteomyelitis | Tuberculosis disease |
| 8833059 | A180 | Tuberculous kyphosis | Tuberculosis disease |
| 8833060 | A180 | Tuberculous lordosis | Tuberculosis disease |
| 8833061 | A180 | Tuberculous scoliosis | Tuberculosis disease |
| 8833799 | A180 | Osteal tuberculosis | Tuberculosis disease |
| 8833830 | A180 | Pelvic tuberculosis | Tuberculosis disease |
| 8835127 | A180 | Deep caries | Tuberculosis disease |
| 8836011 | A180 | Spinal tuberculosis | Tuberculosis disease |
| 8841252 | A180 | Costal caries | Tuberculosis disease |
| 8846558 | A180 | Thoracic vertebrae tuberculosis | Tuberculosis disease |
| 8846570 | A180 | Thoracolumbar tuberculosis | Tuberculosis disease |
| 8846574 | A180 | Cervical vertebrae tuberculosis | Tuberculosis disease |
| 8846977 | A180 | Lumbar tuberculosis | Tuberculosis disease |
| 0160003 | A181 | Renal tuberculosis | Tuberculosis disease |
| 0161008 | A181 | Urinary tract tuberculosis | Tuberculosis disease |
| 0161010 | A181 | Bladder tuberculosis | Tuberculosis disease |
| 0163002 | A181 | Tuberculous prostatitis | Tuberculosis disease |
| 0163005 | A181 | Testicular tuberculosis | Tuberculosis disease |
| 0163007 | A181 | Prostate tuberculosis | Tuberculosis disease |
| 0163009 | A181 | Epididymal tuberculosis | Tuberculosis disease |
| 0164001 | A181 | Vulvar tuberculosis | Tuberculosis disease |
| 0164003 | A181 | Tuberculous salpingitis | Tuberculosis disease |
| 0164004 | A181 | Tuberculous oophoritis | Tuberculosis disease |
| 0169001 | A181 | Genital tuberculosis | Tuberculosis disease |
| 8830622 | A181 | Penile tuberculosis | Tuberculosis disease |
| 8830685 | A181 | Scrotal tuberculosis | Tuberculosis disease |
| 8833053 | A181 | Tuberculous pelvic inflammatory disease | Tuberculosis disease |
| 8833055 | A181 | Tuberculous pyelitis | Tuberculosis disease |
| 8833056 | A181 | Tuberculous pyelonephritis | Tuberculosis disease |
| 8833058 | A181 | Tuberculous vasitis | Tuberculosis disease |
| 8833068 | A181 | Tuberculous pyonephrosis | Tuberculosis disease |
| 8833082 | A181 | Tuberculous ovarian cyst | Tuberculosis disease |
| 8833358 | A181 | Mesenteric tuberculosis | Tuberculosis disease |
| 8834222 | A181 | Uterine tuberculosis | Tuberculosis disease |
| 8835846 | A181 | Tuberculous funiculitis | Tuberculosis disease |
| 8835948 | A181 | Tuberculous epididymitis | Tuberculosis disease |
| 8837426 | A181 | Tuberculous pouch of Douglas | Tuberculosis disease |
| 8838505 | A181 | Ureteral tuberculosis | Tuberculosis disease |
| 8838535 | A181 | Tuberculous bulbourethral gland infection | Tuberculosis disease |
| 8838538 | A181 | Urethral tuberculosis | Tuberculosis disease |
| 8839340 | A181 | Urogenital tuberculosis | Tuberculosis disease |
| 0172005 | A182 | Tuberculous lymphadenitis | Tuberculosis disease |
| 0172020 | A182 | Cervical lymph node tuberculosis | Tuberculosis disease |
| 0149007 | A183 | Tuberculous peritonitis | Tuberculosis disease |
| 0149019 | A183 | Anal tuberculosis | Tuberculosis disease |
| 8830141 | A183 | Sigmoid colon tuberculosis | Tuberculosis disease |
| 8831035 | A183 | Ileal tuberculosis | Tuberculosis disease |
| 8831055 | A183 | Ileocecal tuberculosis | Tuberculosis disease |
| 8832791 | A183 | Jejunal tuberculosis | Tuberculosis disease |
| 8833046 | A183 | Tuberculous diarrhea | Tuberculosis disease |
| 8833054 | A183 | Tuberculous anal fistula | Tuberculosis disease |
| 8833077 | A183 | Tuberculous ascites | Tuberculosis disease |
| 8834759 | A183 | Small intestine tuberculosis | Tuberculosis disease |
| 8835293 | A183 | Duodenal tuberculosis | Tuberculosis disease |
| 8837371 | A183 | Colonic tuberculosis | Tuberculosis disease |
| 8837704 | A183 | Mesenteric lymph node tuberculosis | Tuberculosis disease |
| 8837714 | A183 | Intestinal tuberculosis | Tuberculosis disease |
| 8837781 | A183 | Rectal tuberculosis | Tuberculosis disease |
| 0170006 | A184 | Necrotizing tuberculid | Tuberculosis disease |
| 0170011 | A184 | Lupus vulgaris | Tuberculosis disease |
| 0170013 | A184 | Cutaneous tuberculosis | Tuberculosis disease |
| 0170014 | A184 | Tuberculosis cutis orificialis | Tuberculosis disease |
| 0170015 | A184 | Tuberculous verruca | Tuberculosis disease |
| 0170021 | A184 | Ulcerative lupus | Tuberculosis disease |
| 0170027 | A184 | Sclerotic lupus | Tuberculosis disease |
| 0171004 | A184 | Tuberculid | Tuberculosis disease |
| 8831966 | A184 | Tuberculosis of eyelid | Tuberculosis disease |
| 8833045 | A184 | Tuberculous blepharitis | Tuberculosis disease |
| 8833048 | A184 | Tuberculous nodular erythema | Tuberculosis disease |
| 8839758 | A184 | Abdominal cold abscess | Tuberculosis disease |
| 0173005 | A185 | Tuberculous keratitis | Tuberculosis disease |
| 0173010 | A185 | Tuberculous iridocyclitis | Tuberculosis disease |
| 8831950 | A185 | Ocular tuberculosis | Tuberculosis disease |
| 8833035 | A185 | Tuberculous keratoconjunctivitis | Tuberculosis disease |
| 8833036 | A185 | Tuberculous keratoscleritis | Tuberculosis disease |
| 8833049 | A185 | Tuberculous iritis | Tuberculosis disease |
| 8833078 | A185 | Tuberculous uveitis | Tuberculosis disease |
| 8833080 | A185 | Tuberculous chorioretinitis | Tuberculosis disease |
| 8833081 | A185 | Tuberculous retinitis | Tuberculosis disease |
| 8833175 | A185 | Conjunctival tuberculosis | Tuberculosis disease |
| 8840451 | A185 | Tuberculous choroiditis | Tuberculosis disease |
| 0174001 | A186 | Tuberculous otitis media | Tuberculosis disease |
| 0174002 | A186 | Ear tuberculosis | Tuberculosis disease |
| 8835195 | A186 | Eustachian tube tuberculosis | Tuberculosis disease |
| 8833034 | A187 | Tuberculous Addison's disease | Tuberculosis disease |
| 8833063 | A187 | Tuberculous hypoadrenalism | Tuberculosis disease |
| 8839681 | A187 | Adrenal tuberculosis | Tuberculosis disease |
| 0119013 | A188 | Oral tuberculosis | Tuberculosis disease |
| 0178001 | A188 | Gastric tuberculosis | Tuberculosis disease |
| 8831500 | A188 | Hepatic tuberculosis | Tuberculosis disease |
| 8831840 | A188 | Submandibular tuberculosis | Tuberculosis disease |
| 8832529 | A188 | Thymic tuberculosis | Tuberculosis disease |
| 8832741 | A188 | Muscular tuberculosis | Tuberculosis disease |
| 8832746 | A188 | Fascial tuberculosis | Tuberculosis disease |
| 8833052 | A188 | Tuberculous cardiomyopathy | Tuberculosis disease |
| 8833064 | A188 | Tuberculous arteritis | Tuberculosis disease |
| 8833065 | A188 | Tuberculous endarteritis | Tuberculosis disease |
| 8833070 | A188 | Tuberculous cerebral arteritis | Tuberculosis disease |
| 8833076 | A188 | Tuberculous anemia | Tuberculosis disease |
| 8833369 | A188 | Uvular tuberculosis | Tuberculosis disease |
| 8833411 | A188 | Oral mucosa tuberculosis | Tuberculosis disease |
| 8833469 | A188 | Lip tuberculosis | Tuberculosis disease |
| 8833506 | A188 | Thyroid tuberculosis | Tuberculosis disease |
| 8834850 | A188 | Esophageal tuberculosis | Tuberculosis disease |
| 8834916 | A188 | Myocardial tuberculosis | Tuberculosis disease |
| 8835118 | A188 | Endocardial tuberculosis | Tuberculosis disease |
| 8835135 | A188 | Pericardial tuberculosis | Tuberculosis disease |
| 8836062 | A188 | Fibrocaseous pericarditis | Tuberculosis disease |
| 8837168 | A188 | Gallbladder tuberculosis | Tuberculosis disease |
| 8837421 | A188 | Salivary gland tuberculosis | Tuberculosis disease |
| 0180001 | A192 | Acute miliary tuberculosis | Tuberculosis disease |
| 0189001 | A199 | Miliary tuberculosis | Tuberculosis disease |
| 0189005 | A199 | Cutaneous miliary tuberculosis | Tuberculosis disease |
| 8831063 | A199 | Ulcerative miliary tuberculosis | Tuberculosis disease |
| 8838943 | A199 | Disseminated tuberculosis | Tuberculosis disease |
| 8843639 | B200 | HIV-related non-tuberculous mycobacterial disease | HIV/AIDs |
| 8830096 | B202 | HIV-related cytomegalovirus infection | HIV/AIDs |
| 8849060 | B203 | HIV-related herpes virus infection | HIV/AIDs |
| 8830094 | B204 | HIV-related candidiasis | HIV/AIDs |
| 8850707 | B205 | HIV-related cryptococcosis | HIV/AIDs |
| 8850708 | B205 | HIV-related cryptococcal meningitis | HIV/AIDs |
| 8830092 | B206 | HIV-related Pneumocystis pneumonia | HIV/AIDs |
| 8830091 | B210 | HIV-related Kaposi's sarcoma | HIV/AIDs |
| 8830099 | B211 | HIV-related Burkitt lymphoma | HIV/AIDs |
| 8830100 | B212 | HIV-related non-Hodgkin lymphoma | HIV/AIDs |
| 8830098 | B220 | HIV encephalopathy | HIV/AIDs |
| 8845516 | B220 | HIV-related dementia | HIV/AIDs |
| 8830093 | B221 | HIV-related interstitial pneumonia | HIV/AIDs |
| 8835763 | B222 | Slim disease | HIV/AIDs |
| 8832271 | B230 | Acute HIV infection syndrome | HIV/AIDs |
| 8830097 | B238 | HIV-related nephropathy | HIV/AIDs |
| 8844004 | B238 | HIV-related retinopathy | HIV/AIDs |
| 0798002 | B24 | HIV infection | HIV/AIDs |
| 2793007 | B24 | Acquired immunodeficiency syndrome (AIDS) | HIV/AIDs |
| 2793011 | B24 | AIDS | HIV/AIDs |
| 7712015 | B24 | Neonatal HIV infection | HIV/AIDs |
| 8830055 | B24 | AIDS-related complex | HIV/AIDs |
| 8842156 | B24 | HIV infection | HIV/AIDs |
| 8847287 | B24 | HIV-1 infection | HIV/AIDs |
| 8847288 | B24 | HIV-2 infection | HIV/AIDs |
| 2500001 | E11 | Insulin-resistant diabetes | Diabetes |
| 2500015 | E11 | Type 2 diabetes | Diabetes |
| 8830405 | E11 | Stable diabetes | Diabetes |
| 8835244 | E11 | Young-onset type 2 diabetes | Diabetes |
| 8830041 | E110 | Type 2 diabetic coma | Diabetes |
| 8841689 | E110 | Type 2 diabetes with coma | Diabetes |
| 8845094 | E110 | Type 2 diabetes with hypoglycemic coma | Diabetes |
| 8830040 | E111 | Type 2 diabetic ketoacidosis | Diabetes |
| 8841690 | E111 | Type 2 diabetes with ketoacidosis | Diabetes |
| 8845073 | E111 | Type 2 diabetes with acidosis | Diabetes |
| 8845074 | E111 | Type 2 diabetes with ketonemia | Diabetes |
| 8849058 | E111 | Type 2 diabetes with ketosis | Diabetes |
| 8830042 | E112 | Type 2 diabetic nephropathy | Diabetes |
| 8841691 | E112 | Type 2 diabetes with renal complications | Diabetes |
| 8843991 | E112 | Type 2 diabetic nephropathy stage 1 | Diabetes |
| 8843992 | E112 | Type 2 diabetic nephropathy stage 2 | Diabetes |
| 8843993 | E112 | Type 2 diabetic nephropathy stage 3 | Diabetes |
| 8843994 | E112 | Type 2 diabetic nephropathy stage 3A | Diabetes |
| 8843995 | E112 | Type 2 diabetic nephropathy stage 3B | Diabetes |
| 8843996 | E112 | Type 2 diabetic nephropathy stage 4 | Diabetes |
| 8843997 | E112 | Type 2 diabetic nephropathy stage 5 | Diabetes |
| 8845087 | E112 | Type 2 diabetes with renal sclerosis | Diabetes |
| 8845088 | E112 | Type 2 diabetes with renal failure | Diabetes |
| 8830045 | E113 | Type 2 diabetic retinopathy | Diabetes |
| 8841692 | E113 | Type 2 diabetes with ophthalmic complications | Diabetes |
| 8843990 | E113 | Type 2 diabetes with macular edema | Diabetes |
| 8844347 | E113 | Type 2 diabetic cataract | Diabetes |
| 8844537 | E113 | Proliferative diabetic retinopathy in type 2 diabetes | Diabetes |
| 8845072 | E113 | Type 2 diabetes with maculopathy | Diabetes |
| 8845078 | E113 | Type 2 diabetes with ocular palsy | Diabetes |
| 8845082 | E113 | Type 2 diabetes with iritis | Diabetes |
| 8845093 | E113 | Type 2 diabetes with central retinopathy | Diabetes |
| 8830043 | E114 | Type 2 diabetic neuropathy | Diabetes |
| 8830044 | E114 | Type 2 diabetic myopathy | Diabetes |
| 8841693 | E114 | Type 2 diabetes with neurological complications | Diabetes |
| 8845079 | E114 | Type 2 diabetes with muscular atrophy | Diabetes |
| 8845084 | E114 | Type 2 diabetes with neurogenic bladder | Diabetes |
| 8845085 | E114 | Type 2 diabetes with neuralgia | Diabetes |
| 8845086 | E114 | Type 2 diabetes with autonomic neuropathy | Diabetes |
| 8845091 | E114 | Type 2 diabetes with polyneuropathy | Diabetes |
| 8845092 | E114 | Type 2 diabetes with mononeuropathy | Diabetes |
| 8845100 | E114 | Type 2 diabetes with peripheral neuropathy | Diabetes |
| 8841694 | E115 | Type 2 diabetes with peripheral circulatory complications | Diabetes |
| 8843106 | E115 | Type 2 diabetes with gangrene | Diabetes |
| 8845075 | E115 | Type 2 diabetes with ulcer | Diabetes |
| 8845080 | E115 | Type 2 diabetes with vascular complications | Diabetes |
| 8845095 | E115 | Type 2 diabetes with arteriosclerosis | Diabetes |
| 8845096 | E115 | Type 2 diabetes with arterial occlusion | Diabetes |
| 8845098 | E115 | Type 2 diabetes with peripheral angiopathy | Diabetes |
| 8845099 | E115 | Type 2 diabetes with peripheral vascular disorder | Diabetes |
| 8841695 | E116 | Type 2 diabetes with arthropathy | Diabetes |
| 8841696 | E116 | Type 2 diabetes with multiple complications | Diabetes |
| 8844628 | E116 | Type 2 diabetes with bullae | Diabetes |
| 8844629 | E116 | Type 2 diabetes with scleredema | Diabetes |
| 8845076 | E116 | Type 2 diabetes with hepatic disorder | Diabetes |
| 8845077 | E116 | Type 2 diabetes with arthralgia | Diabetes |
| 8845081 | E116 | Type 2 diabetes with hypercholesterolemia | Diabetes |
| 8845083 | E116 | Type 2 diabetes with osteopathy | Diabetes |
| 8845089 | E116 | Type 2 diabetes with mental disorder | Diabetes |
| 8845090 | E116 | Type 2 diabetes with pruritus | Diabetes |
| 8845097 | E116 | Type 2 diabetes with dermatopathy | Diabetes |
| 8848108 | E116 | Type 2 diabetes with gastrointestinal disorder | Diabetes |
| 8849558 | E116 | Type 2 diabetes with hyperosmolar hyperglycemic syndrome | Diabetes |
| 8841697 | E117 | Type 2 diabetes with multiple diabetic complications | Diabetes |
| 8841698 | E119 | Type 2 diabetes without complications | Diabetes |
| 2500014 | E10 | Type 1 diabetes | Diabetes |
| 2500027 | E10 | Unstable diabetes | Diabetes |
| 8844022 | E10 | Slowly progressive Type 1 diabetes | Diabetes |
| 8830030 | E100 | Type 1 diabetes with coma | Diabetes |
| 8841679 | E100 | Type 1 diabetes with coma | Diabetes |
| 8844026 | E100 | Slowly progressive Type 1 diabetes with coma | Diabetes |
| 8845065 | E100 | Type 1 diabetes with hypoglycemic coma | Diabetes |
| 8830028 | E101 | Type 1 diabetic ketoacidosis | Diabetes |
| 8841680 | E101 | Type 1 diabetes with ketoacidosis | Diabetes |
| 8844025 | E101 | Slowly progressive Type 1 diabetes with ketoacidosis | Diabetes |
| 8844045 | E101 | Fulminant Type 1 diabetes | Diabetes |
| 8845044 | E101 | Type 1 diabetes with acidosis | Diabetes |
| 8845045 | E101 | Type 1 diabetes with ketonemia | Diabetes |
| 8849056 | E101 | Type 1 diabetes with ketosis | Diabetes |
| 8830031 | E102 | Type 1 diabetic nephropathy | Diabetes |
| 8841681 | E102 | Type 1 diabetes with renal complications | Diabetes |
| 8843983 | E102 | Type 1 diabetic nephropathy stage 1 | Diabetes |
| 8843984 | E102 | Type 1 diabetic nephropathy stage 2 | Diabetes |
| 8843985 | E102 | Type 1 diabetic nephropathy stage 3 | Diabetes |
| 8843986 | E102 | Type 1 diabetic nephropathy stage 3A | Diabetes |
| 8843987 | E102 | Type 1 diabetic nephropathy stage 3B | Diabetes |
| 8843988 | E102 | Type 1 diabetic nephropathy stage 4 | Diabetes |
| 8843989 | E102 | Type 1 diabetic nephropathy stage 5 | Diabetes |
| 8844028 | E102 | Slowly progressive Type 1 diabetes with renal complications | Diabetes |
| 8845058 | E102 | Type 1 diabetes with renal sclerosis | Diabetes |
| 8845059 | E102 | Type 1 diabetes with renal failure | Diabetes |
| 8830033 | E103 | Type 1 diabetic retinopathy | Diabetes |
| 8841682 | E103 | Type 1 diabetes with ophthalmic complications | Diabetes |
| 8843982 | E103 | Type 1 diabetes with macular edema | Diabetes |
| 8844024 | E103 | Slowly progressive Type 1 diabetes with ophthalmic complications | Diabetes |
| 8844346 | E103 | Type 1 diabetic cataract | Diabetes |
| 8844536 | E103 | Proliferative diabetic retinopathy in Type 1 diabetes | Diabetes |
| 8845043 | E103 | Type 1 diabetes with maculopathy | Diabetes |
| 8845049 | E103 | Type 1 diabetes with ocular palsy | Diabetes |
| 8845053 | E103 | Type 1 diabetes with iritis | Diabetes |
| 8845064 | E103 | Type 1 diabetes with central retinopathy | Diabetes |
| 8830032 | E104 | Type 1 diabetic neuropathy | Diabetes |
| 8841683 | E104 | Type 1 diabetes with neurological complications | Diabetes |
| 8844027 | E104 | Slowly progressive Type 1 diabetes with neurological complications | Diabetes |
| 8845050 | E104 | Type 1 diabetes with muscular atrophy | Diabetes |
| 8845055 | E104 | Type 1 diabetes with neurogenic bladder | Diabetes |
| 8845056 | E104 | Type 1 diabetes with neuralgia | Diabetes |
| 8845057 | E104 | Type 1 diabetes with autonomic neuropathy | Diabetes |
| 8845062 | E104 | Type 1 diabetes with polyneuropathy | Diabetes |
| 8845063 | E104 | Type 1 diabetes with mononeuropathy | Diabetes |
| 8845071 | E104 | Type 1 diabetes with peripheral neuropathy | Diabetes |
| 8841684 | E105 | Type 1 diabetes with peripheral circulatory complications | Diabetes |
| 8843105 | E105 | Type 1 diabetes with gangrene | Diabetes |
| 8844031 | E105 | Slowly progressive Type 1 diabetes with peripheral circulatory complications | Diabetes |
| 8845046 | E105 | Type 1 diabetes with ulcer | Diabetes |
| 8845051 | E105 | Type 1 diabetes with vascular complications | Diabetes |
| 8845066 | E105 | Type 1 diabetes with arteriosclerosis | Diabetes |
| 8845067 | E105 | Type 1 diabetes with arterial occlusion | Diabetes |
| 8845069 | E105 | Type 1 diabetes with peripheral angiopathy | Diabetes |
| 8845070 | E105 | Type 1 diabetes with peripheral vascular disorder | Diabetes |
| 8841685 | E106 | Type 1 diabetes with arthropathy | Diabetes |
| 8841686 | E106 | Type 1 diabetes with multiple complications | Diabetes |
| 8844023 | E106 | Slowly progressive Type 1 diabetes with arthropathy | Diabetes |
| 8844626 | E106 | Type 1 diabetes with bullae | Diabetes |
| 8844627 | E106 | Type 1 diabetes with scleredema | Diabetes |
| 8845047 | E106 | Type 1 diabetes with hepatic disorder | Diabetes |
| 8845048 | E106 | Type 1 diabetes with arthralgia | Diabetes |
| 8845052 | E106 | Type 1 diabetes with hypercholesterolemia | Diabetes |
| 8845054 | E106 | Type 1 diabetes with osteopathy | Diabetes |
| 8845060 | E106 | Type 1 diabetes with mental disorder | Diabetes |
| 8845061 | E106 | Type 1 diabetes with pruritus | Diabetes |
| 8845068 | E106 | Type 1 diabetes with skin disorder | Diabetes |
| 8845842 | E106 | Type 1 diabetes with gastrointestinal disorder | Diabetes |
| 8849557 | E106 | Type 1 diabetes with hyperglycemic hyperosmolar syndrome | Diabetes |
| 8841687 | E107 | Type 1 diabetes with multiple diabetic complications | Diabetes |
| 8844029 | E107 | Slowly progressive Type 1 diabetes with multiple diabetic complications | Diabetes |
| 8841688 | E109 | Type 1 diabetes without complications | Diabetes |
| 8844030 | E109 | Slowly progressive Type 1 diabetes without complications | Diabetes |
